# Achieving eco-innovative smart glass design with the integration of opinion mining, QFD and TRIZ

**DOI:** 10.1038/s41598-024-58867-1

**Published:** 2024-04-29

**Authors:** C. K. M. Lee, Y. P. Tsang, W. W. Chong, Y. S. Au, J. Y. Liang

**Affiliations:** 1 The Hong Kong Polytechnic University, Kowloon, Hong Kong; 2 Laboratory for Artificial Intelligence in Design, New Territories, Hong Kong

**Keywords:** Smart glasses, Sustainable design, Eco-innovation, Text mining, Quality function deployment, Theory of inventive problem solving, Engineering, Information technology

## Abstract

Modern consumption patterns lead to massive waste, which poses challenges in storage and highlights the urgent need for more sustainable product development. Customer feedback on products plays a crucial role in product design, yet previous studies overlooked these invaluable insights. In response, this study introduces a novel systematic methodology that integrates the strengths of text mining, Quality Function Deployment (QFD), and the Theory of Inventive Problem Solving (TRIZ). Text mining techniques are utilized to extract customer requirements from online platforms, while QFD is used to translate these requirements into technical specifications. By integrating the contradiction matrix from TRIZ theory with the triptych, technical conflicts are resolved. The design process for next-generation smart glasses is employed as an illustrative case to validate the proposed integrated innovation design approach. Analytical outcomes suggest that the introduced methodology can effectively address sustainable product design challenges and sets the stage for future advancements in smart glasses.

## Introduction

With the growing popularity of smart devices, Augmented Reality (AR) technology has undergone significant development and established itself as a crucial domain for these intelligent devices. Augmented Reality (AR) integrates digital elements with the physical world, creating an interactive environment where real and virtual components coexist. This technology enhances real-world surroundings by overlaying them with digital information and interfaces^1^. According to a market analysis report by Grand View Research^2^, the market value of augmented reality was at $38.56 billion in 2022 and is expected to grow at a compound annual growth rate (CAGR) of 39.8% from 2023 to 2030. Smart glasses, standing out as a promising major platform for AR, are on the verge of becoming the next leading smart devices in the upcoming years. Smart glasses are wearable devices that integrate a see-through optical display, typically within the user's field of vision, to facilitate the merging of the physical environment with virtual elements.

The surge of interest in smart glasses from leading technology leaders like Google LLC, Microsoft Corporation, Apple, Inc., and others, who are exploring diverse applications for smart glasses, is creating new opportunities in this field^3^.

However, this boom in new smart glasses has also contributed to a rise in e-waste^4^, presenting manufacturers with a new challenge: How to extend the lifespan of smart products during the maturity phase to avoid them being phased out of the market?

Sustainable Design offers a solution to these problems by specializing on the development of products, services, and systems that promote sustainable development^5^. This approach considers the entire lifecycle of a product, from design and manufacturing to use and eventual disposal, with an aim to minimize ecological footprints and promote responsible resource usage. Sustainable design tools, particularly QFD and TRIZ, have been explored and applied to design process by engineers and designers to develop more environmentally friendly product designs^6–8^. Their combine application has shown promising outcomes in facilitating sustainable design strategies.

However, the application of sustainable design in smart glasses remains limited. Most research focuses on utilizing smart glasses for efficient sustainable applications, like advancing sustainable education^9–11^. There is limited literature that addresses the sustainable design aspects of smart glasses as a product^12^.

In addition, traditional research approaches in sustainable design often overlook the evolving customer preferences from product reviews, as it is challenging for them to leverage the vast volume of unstructured data available on the digital platforms^13^. Incorporating customer feedback into product design is crucial for gaining insights into user experiences and expectations^14^. This approach not only identifies areas for innovation and improvement but also influences production costs and market strategies. By integrating these insights into the design process, products become more technologically advanced and user-centric, thus extending the lifespan of products and furthering sustainability goals. In summary, three research questions (RQs) are formulated as follows:

### RQ1: 

How can the customer feedback from digital platforms be identified and analyzed to improve the design of smart glasses?

### RQ2: 

What are the key factors that influence customer satisfaction with smart glasses, and how can these insights be integrated into QFD and TRIZ methodologies to guide the design of next-generation smart glasses for more customer satisfaction and sustainability?

### RQ3: 

What is the next-generation smart glasses design based on latest customer requirements?

Text mining techniques (also known as Opinion Mining), through automated sentiment analysis and topic extraction, could enable designers and manufacturers to identify and respond to real-time issues and evolving trends in customer feedback^15^. Numerous studies highlight the integration of text mining techniques into the design process as an efficient and insightful approach^16–18^. Traditional methods like Face-to-Face Interviews, Telephone Surveys, and Paper and Pencil Recording are time-consuming and not suited for processing large volumes of unstructured data^19^. Text mining poses the ability of analyzing extensive textual data in real-time, which could facilitate the rapid and efficient collection of users feedback and foster a more responsive and user-centered approach to product design.

Therefore, a systematic approach for supporting sustainable product design based on text mining, Quality Function Deployment (QFD), and the Theory of Inventive Problem Solving (TRIZ) is proposed. The proposed methodology aims to evaluate customer feedback from online product reviews and identify the most important design attributes based on that feedback.

The process starts by web scraping to extract product reviews from online platforms. The mined text data then undergoes pre-processing using the Natural Language Toolkit (NLTK). Following by this, TextRank^20^, a text mining algorithm, is employed to identify frequently occurring keywords in product reviews and capture what consumers feel and focus on about the product. The QFD technique^21^ is applied to analyse customer requirements from the extracted textual data and transform them into technical specifications through a structured relationship matrix^22^. Furthermore, an assessment of the correlation between customer needs and corresponding technical solutions is conducted to identify product attributes that resonate with potential customers and match with design objectives. Based on the technical specifications and customer requirements derived from QFD analysis, TRIZ and Triptych^23^ are combined to identify and address technical contradictions that might arise during the product design process. The contradiction matrix can categorize these technical contradictions and propose standardized solutions for each pair of contradictions, known as ‘inventive principles’. By leveraging these recommended principles, designers can systematically address design contradictions, thereby meeting the diverse requirements of customers and amplifying the product's popularity in the marketplace.

The proposed system offers a multi-faceted advantage in sustainable design by guiding designers and manufacturers toward understanding market demands. The systematic approach transforms sustainability from a mere buzzword into a vital product attribute, which aids in addressing the challenges of new customer requirements and technical disparities. To verify the feasibility and effectiveness of the proposed system, a case study on smart glasses was conducted. Eco-innovation guidelines for the next-generation smart glasses were generated through the proposed system to meet both consumer preferences and environmental standards. In summary, by fusing customer insights, technical expertise, and innovative solutions, the proposed system emphasizes sustainability across the entire supply chain of innovative tech products.

The rest of this study is organized as follows: Section "Literature review" reviews the literature on sustainable design thinking, text mining approaches, and outline the motivation of this study. Section "Methodology" presents the research methodology for identifying customer requirements from online reviews to facilitate the customer-oriented product design. Smart glasses are considered as an example to validate the proposed system in Section "Case analysis", while research findings and suggested features for future smart glasses design are discussed in Section "Results and discussion". Finally, the conclusion of this study is drawn in Section "Conclusions".

## Literature review

Sustainable design refers to the design concept that considers the whole product and service design process. It is described as recognizing the symbiotic relationship between enterprises, consumers, and the ecological environment^24^.

Incorporating sustainable design thinking during the initial design and development stage is essential^25^. As noted by the European Commission^26^, over 80% of a product's sustainability is established during this phase, with each decision made by the product development team being crucial for achieving sustainable outcomes^27^. Steenis et al.^28^ further highlighted that integrating sustainability into redesign efforts not only boosts consumer satisfaction but also increases purchase intentions.

Key principles of sustainable design, such as Life Cycle Thinking, Circular Economy, Material Selection, Energy Efficiency, Minimizing Waste, User-Centered Design, Biomimicry^29^, etc., have been explored and implemented across various industries. For instance, in construction^30–32^, packaging^33^, fashion^34^, transportation^35^, etc.

Some design techniques, such as QFD and TRIZ, have proven successful in incorporating sustainable design concepts into product development.

Quality Function Deployment (QFD)^21^ is a tool in quality management and product development that formulates connections between customer requirements, often known as the Voice of the Customer, and the attributes and specifications of a product or service. It is used to align design and manufacturing decisions with customer needs by creating a House of Quality (HOQ) that links customer requirements to the product or service features and specifications. QFD assists designers in understanding customer expectations, improving product or service quality, reducing design and production errors, and ultimately enhancing customer satisfaction.

TRIZ theory, developed by Genrich Altshuller^23^, serves as an approach to help designers quickly identify problems and utilize inventive principles to find optimal solutions. The core of TRIZ involves the detection of contradictions among different design parameters, where one aspect requires improvement while enhancing it may potentially worsen another. This process leads to the identification of relevant inventive principles, which offer partial solutions to the problem. TRIZ comprises a total of 40 inventive principles, which were summarized by Genrich Altshuller^23^ and his peers from an extensive analysis of patent data. TRIZ demonstrates remarkable adaptability and is applicable across diverse domains, including engineering, product design, and technology development. It assists designers in selecting the optimal methods to enhance the quality and effectiveness of innovation.

The integration of QFD, TRIZ and other methodologies in the design process has been explored by many scholars. For instance, Chunjing, Wu, Chen, and Ling^36^ introduced an integrated innovation design model based on KANO, QFD, and TRIZ theories for rewinding machine design. Hameed et al.^37^ combine FMEA, QFD, TRIZ, LCA, and fuzzy TOPSIS methodologies to redesign a pressure relief valve (PRV) in an economic, sustainable, and innovative way. Li et al.^38^ proposed a design methodology that incorporates input from VTS personnel, QFD, TRIZ, and software quality characteristics to improve the interaction between alarm systems and operators.

However, these traditional methods often struggle to adapt to changing customer demands. With individuals now openly expressing their views on products and services, the task of gathering the vast amount of unstructured data from digital platforms presents a challenge for designers and manufacturers. This customer feedback could have an impact on the design process, the production costs associated with the product, and customer satisfaction levels with the products.

Applying text mining techniques provides an effective solution to address the challenges posed by changing customer demands and the abundance of unstructured digital data^39^. Text mining enables designers and manufacturers to efficiently analyse customer feedback, automating sentiment analysis and topic extraction to identify emerging trends and issues in real-time^15^. This not only aids in adapting product designs instantly but also optimizes production processes and cost management. By measuring customer sentiment and addressing concerns, text mining enhances overall customer satisfaction and associates products with market demands. TextRank, introduced by Mihalcea and Tarau^40^ in 2004, offers a frequently used approach for summarizing text without the need for prior linguistic or domain knowledge^41^. It is language-agnostic, focusing on word consistency, eliminating the need for language-specific technologies. While it may face variations in sentence splitting among languages, it remains a well-developed and accessible system for developers interested in leveraging its advantages for extraction-based summarization^42^.

After analysing the three methods mentioned above, it becomes evident that each of them plays a distinct role and function within the product design process. This study proposes a systematic approach for product design that integrates text mining, QFD, and TRIZ methods. It begins with gathering product reviews through web scraping and then utilizes NLTK and TextRank to analyse consumer feedback. QFD is employed to convert customer needs into technical specifications, and the relationship between these needs and technical solutions is assessed to identify suitable product features. TRIZ and Triptych help resolve technical conflicts using a contradiction matrix and inventive principles. This systematic approach can be applied to the product design process for smart devices and offers the benefits of improved alignment with customer requirements, streamlined conflict resolution, and enhanced market competitiveness.

## Methodology

**Figure 1:**
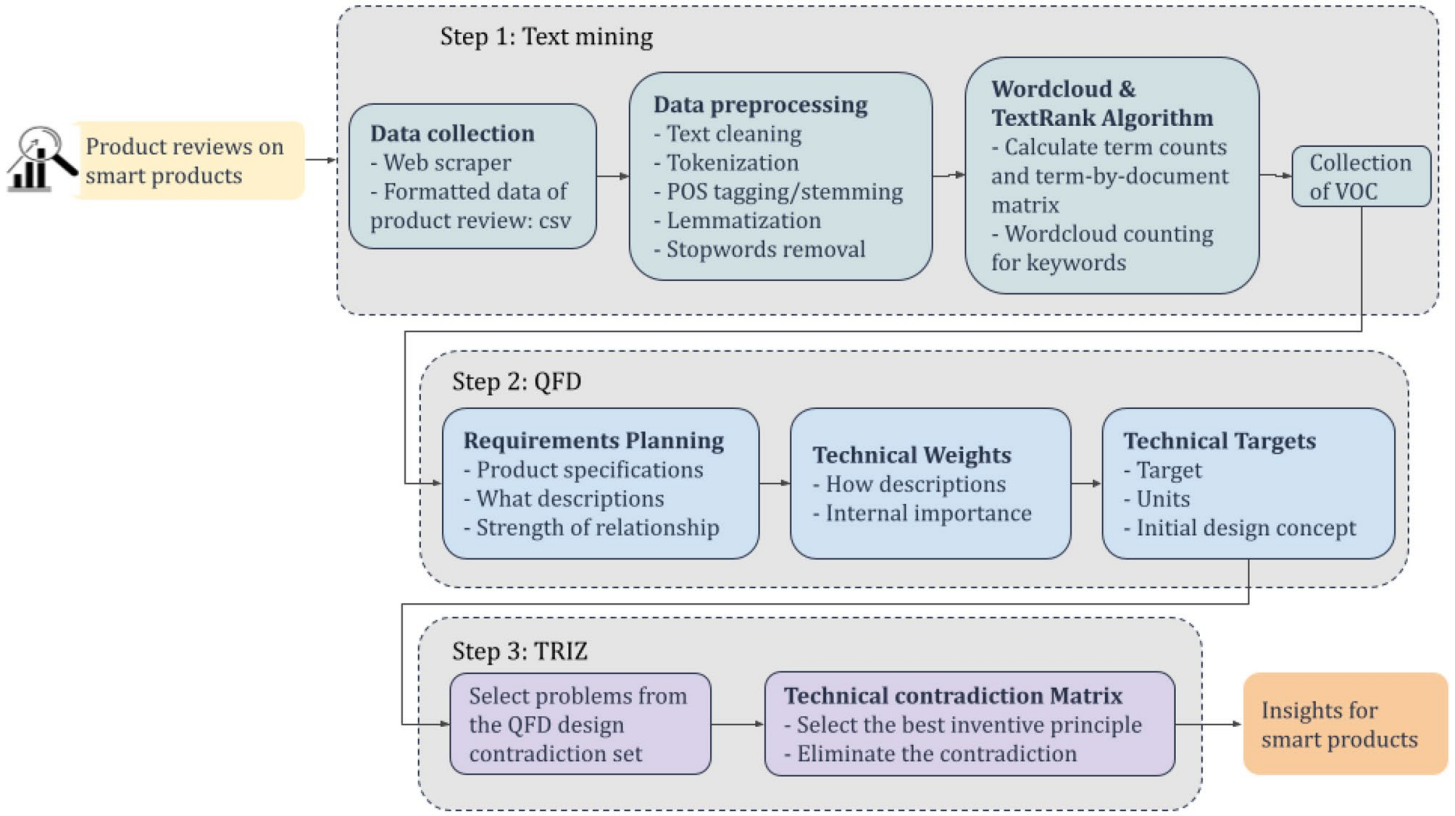
Product design framework with online review.

The proposed methodology consists of three processes for examining customer feedback from online product reviews and evaluating the functions of recommended design attributes: text mining, QFD and TRIZ approaches. The framework of the proposed system is depicted in Fig. 1.

### Step 1: text mining

The text mining technique is applied to extract valuable patterns and insights from vast quantities of unstructured customer reviews. It aids in understanding customer sentiments and primary concerns about products so as to make it crucial for identifying key features for improvement.

#### Data collection

Web scraping is a technology used to access and retrieve massive amounts of data from public websites^43^. It aims to convert unstructured data into structured information to make it useful and actionable^44^. In this study, a web scraper was built to collect information about customer reviews from an online platform for further analysis by using the library Selenium, provided by Python.

#### Data pre-processing

The mined text data then pre-processed using the Natural Language Toolkit (NLTK), which includes text cleaning, tokenization, part-of-speech tagging/stemming, lemmatization, and the stop words removal. Firstly, during the tokenization, since the product reviews containing irrelevant or noisy input like symbols, special characters, illustrations, and issues with cases, several procedures were taken to ensure an accurate result, including the removal of special characters, URLs, and some identical and repeated comments. The stemming process was also utilized to converts the inflected words to their root form. Secondly, part-of-speech (POS) tagging breaks down a phrase into its individual components, which includes a list of words or tuples accompanied by identifiers indicating the word type, such as a noun or verb. Thirdly, lemmatization is the process of appropriately utilizing the vocabulary and morphological interpretation of words, intending to eliminate only inflectional ends and return to the base or dictionary form of a word, referred to as the lemma. For example, turning “listening”, “listened” and “listen” into the basic form of “listen”. Lastly, stop words are the most commonly used words in any language (such as articles, prepositions, pronouns, and conjunctions) that provide little information to the text and must be filtered out before natural language processing. For instance, the words "the", "a", and "an".

Table 1 shows an example of data pre-processing of an extracted comment. The term “worst” has been lemmatized back to its base form of “bad”. The frequently found word “the” was eliminated during the removal of stop words. A collection of specified stop words was also defined in this case. For example, one of the all-too-common words would be “glasses”.

**Table 1:** Example of data pre-processing of an extracted comment.

“Probably the worst gadget ever”	
Tokenization	['Probably', 'the', 'worst', 'gadget', 'ever']
POS tagging	[('Probably', 'RB'), ('the', 'DT'), ('worst', 'JJS'), ('gadget', 'NN'), ('ever', 'RB')]
Lemmatization	['probably', 'the', 'bad', 'gadget', 'ever']
Stop words removal	['probably', 'bad', 'gadget', 'ever']

#### Mining process: Wordcloud and TextRank algorithm

Word clouds are a quicker way to evaluate text retrieved from extracted comments than coding since they decompose the text into component words and calculate their frequency of recurrence within the body of text^45^. Counting the frequency of keywords within the product review, the word cloud provides an overview of the function items and features being discussed.

Following by this, TextRank^40^ is employed for text mining. TextRank is an efficient text mining technique designed for extracting key content from vast textual data. It is a graph-based ranking algorithm that can split textual content, such as articles or product reviews, into individual sentences. Then, it breaks the sentences into smaller phrases and formulates a sparse matrix to clearly lists the words in each phrase or sentence and how often they appear^46^. Different from the traditional co-occurrence relationships, TextRank utilizes similarity scores to determine the importance of each word. This may help in identifying common keywords in product reviews and reveal the consumer sentiments and priorities on products. There are five steps in the TextRank algorithm:

i. Text Segmentation and Part-of-Speech Filtering:
  The target text ***T*** is segmented into sentences ***S***_***1***_**, *S***_***2***_**, …, S**_***m***_. Each sentence ***S***_***i***_, a subset of ***T***, undergoes a filtering process where stop words and words not belonging to a specific part-of-speech are removed. This results in a representation **S**_**i**_** = [t**_**i,1**_**, t**_**i,2**_**, …, t**_**i,n**_**]**, where ***t***_***i,j***_ represents candidate keywords that annotate the word segmentation and filtering process.
ii. Candidate Word Graph Construction:
  Hence, the candidate word graph **G = (V, E)** is constructed. Here, the value **V** refers to the vertex set formed of the candidate words from the previous step, while **E** refers to the subset of V × V’s edge set. Edges are established between nodes based on co-occurrence within a window of length **K**, where **K** refers to the window size and indicates the maximum allowable word count for co-occurrence.
iii. Node Weight Calculation:
  The weight of each node in the candidate word graph is computed iteratively using Eq. (1) until the computation result converges^47^.
  1 $$WS\left({v}_{i}\right)=\left(1-d\right)+d{\sum }_{{v}_{j}\in In({v}_{i})}\frac{{w}_{ji}}{\sum_{{v}_{k}\in {\text{Out}}({v}_{i})}{w}_{jk}}WS\left({v}_{i}\right)$$
  In Eq. (1), **In(vi)** denotes the set of nodes that connect to node vi, and the damping factor **d ∈ [0,1]** is s akin to the random walk probability utilized in the PageRank algorithm. This factor was originally introduced to prevent pages without external links from impeding user navigation, and its typical value is 0.85. If a candidate word graph node's failure rate is less than a set limit value, the node is regarded to have attained convergence; this limit value is often set at 0.0001.
iv. Transition Probability:
  From the traditional TextRank algorithm, as shown in Eq. (2), the transition probability, ***p (v***_***j***_*** ⟶ v***_***i***_***)***, denotes the probability of jumping from node ***v***_***j***_ to node ***v***_***i***_.
  2 $$p\left({v}_{j}\to {v}_{i}\right)=\left\{\begin{array}{c}\begin{array}{cc}\frac{{w}_{ji}}{\sum_{{v}_{k}\in {\text{Out}}({v}_{j})}{w}_{jk}}& if \exists ({v}_{j}\to {v}_{i})\in E \end{array}\\ \begin{array}{cc}0,& {\text{Otherwise}}\end{array}\end{array}\right.$$
  In Eq. (2), ***out (v***_***j***_***)*** denotes the set of nodes referred to by ***v***_***j***_**,** and ***w***_***ji***_ denotes the edge weight from node ***w***_***j***_ to node ***w***_***i***_, which is calculated by the co-occurrence of two words in the conventional algorithm.
v. Keyword Extraction:
  Finally, the node weights are sorted in reverse order, and the first ***K***-word in the target text is utilized as the keyword, where ***K*** is the desired number of keywords for extraction.

In summary, this step facilitates the extraction of key customer insights, including the most frequently discussed product features, customer preferences, and commonly mentioned issues in product reviews. The application of TextRank algorithm and the visualization of text data via word clouds assists in identifying important insights into current customer preferences and expectations for smart products, which lays the groundwork for further analysis in next steps.

### Step 2: quality function deployment

The Quality Function Deployment (QFD) technique^21^ serves as a bridge to convert identified consumer demands into technical specifications. It ensures that products are not only functionally robust but also resonate with the target customer, thus achieving a harmonious balance between technical feasibility and consumer desirability.

The QFD process employs specific terms such as the House of Quality (HOQ), WHATs, and HOWs. The HOQ outlines the steps required when implementing QFD. "WHATs" represent essential customer requirements, often conveyed in imprecise language, and serve as the primary input to the HOQ. "HOWs" denote the technical design characteristics that address these requirements. During the requirements planning phase, customer feedback is gathered through a text mining process. This information aids in constructing the relationship matrix, which depicts the connection between product attributes and customer needs. After identifying the technical specifications (WHATs) and the necessary manufacturing procedures (HOWs), experts assess the strength of their association using technical weights. This relationship is typically rated on a scale of 1 (low), 3 (medium), and 9 (high), with a dash indicating no connection. Technical targets, aligned with the identified product attributes, are then established to encompass target descriptions, units, and preliminary design concepts.

In this step, the customer needs for smart products, identified through text mining in Step 1, are translated into specific technical specifications. A list of product features prioritized based on customer feedback is conducted through QFD methodology, which helps in understanding which aspects of smart glasses are most crucial to consumers and need to be focused on in the design.

### Step 3: theory of inventive problem solving

TRIZ^23^ is utilized to identify contradictions and challenges in the design process. By applying the inventive principles of TRIZ to these design conflicts, innovative design ideas for products are generated. Not only does TRIZ provide solutions for the present, but it also offers strategic insights for future sustainable design and development initiatives.

To derive valuable insights from TRIZ, a series of systematic steps is conducted. First, after employing QFD technique, both customer and technical requirements are identified. The technical contradiction matrix of TRIZ serves a dual purpose: it helps in identifying the technical conflict and translates it into a mix of the 39 design parameters. Once a contradiction is identified, the design parameters of the product that have been enhanced or regressed are determined. Finally, it aids in selecting the most inventive principles tailored for addressing the contradiction. In addressing these technical contradictions, it's imperative to ascertain the aspects of the conflict that need emphasis and those that should be subdued. The most feasible solution is then integrated into product development practices, relying heavily on these principles.

In this step, design contradictions associated with the prioritized product features found in the QFD process could be identified using TRIZ contradiction matrix, and these specific contradictions would be resolved by developing innovative design solutions and alternative strategies utilizing TRIZ inventive principles. This approach aids in improving the functionality of smart products while also considering potential future design challenges.

## Case analysis

As mentioned before, the growing interest in smart devices indicates a trend in the electronics industry towards rapid product evolution and frequent new releases^48^. However, this boom also symbolizes challenges, especially the increase in e-waste. Therefore, smart glasses, as emerging products of smart devices, consider as a case study to assess if our proposed system can investigate the relationship between consumer needs and product design and provide eco-innovative solutions on extending product life and reducing obsolescence.

Smart glasses are now focused on the enterprise market, and the glasses experience did not result in a large-scale consumer ecology. The main reason smart glasses are not widely used in the market at this time is that customers are dissatisfied with the design of smart glasses, rendering them unsuitable for continuous use. For example, the smart glasses that have been officially released on the market, such as Microsoft's Hololens and Lenovo's ThinkReality, are different from ordinary glasses in daily use. The design of the clunky glasses falls short of the thin and sleek look that consumers expect. Meanwhile, there is a lack of strategic design and development of systematic sustainable solutions in the product market. Hence, producing novel and innovative smart glasses for consumers has huge market potential.

### Data collection and data pre-processing

**Figure 2:**
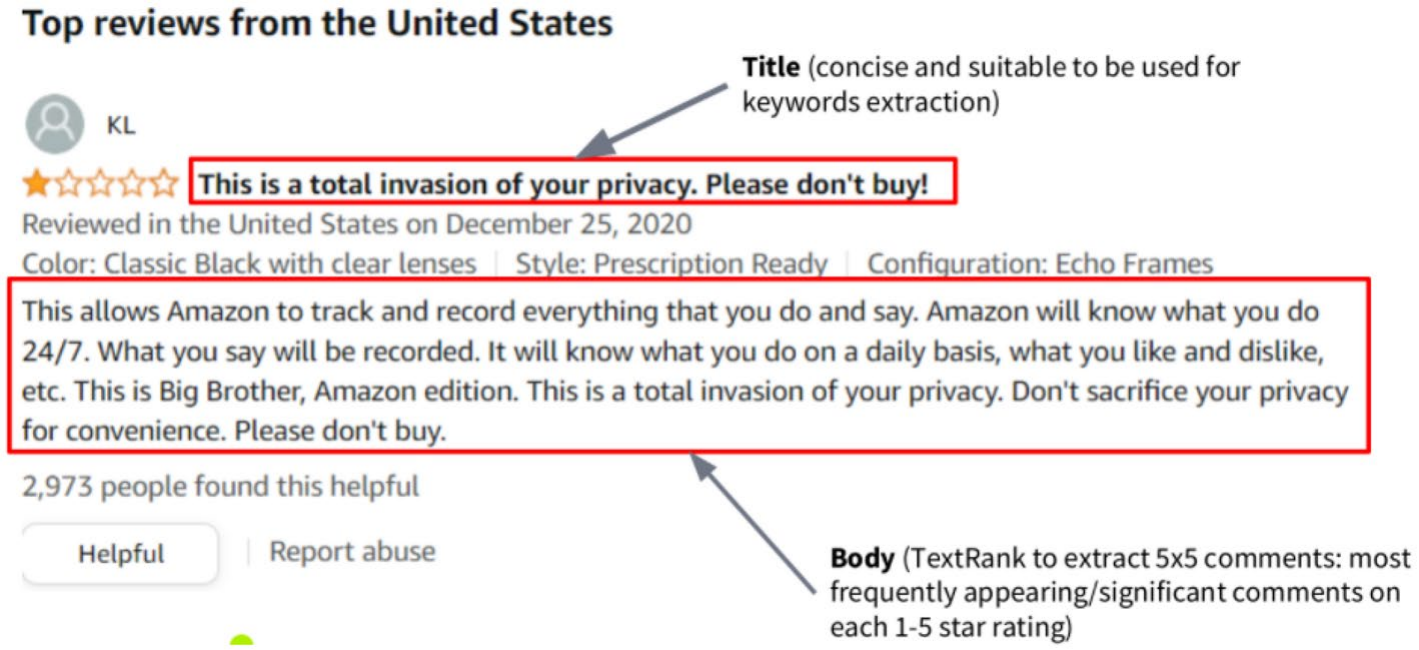
An example of a product review for smart glasses.

Product reviews on smart glasses from Amazon between 2020–2021 were extracted and saved in a CSV format. There were 1997 reviews for 60 different models of smart glasses. These smart glasses not only possess augmented reality features but also integrate the functionalities of sunglasses and headphones, as seen in renowned brands like Epson and MAD Gaze. Figure 2 shows an example of a product review for smart glasses. To better analyse consumers’ feedback, the title column of the reviews was considered for keyword extraction as it typically encapsulates the primary views of customers. Furthermore, the body column from 10 comments was extracted to represent the most frequently appearing or noteworthy comments within each 1–5 star rating category.

**Figure 3:**
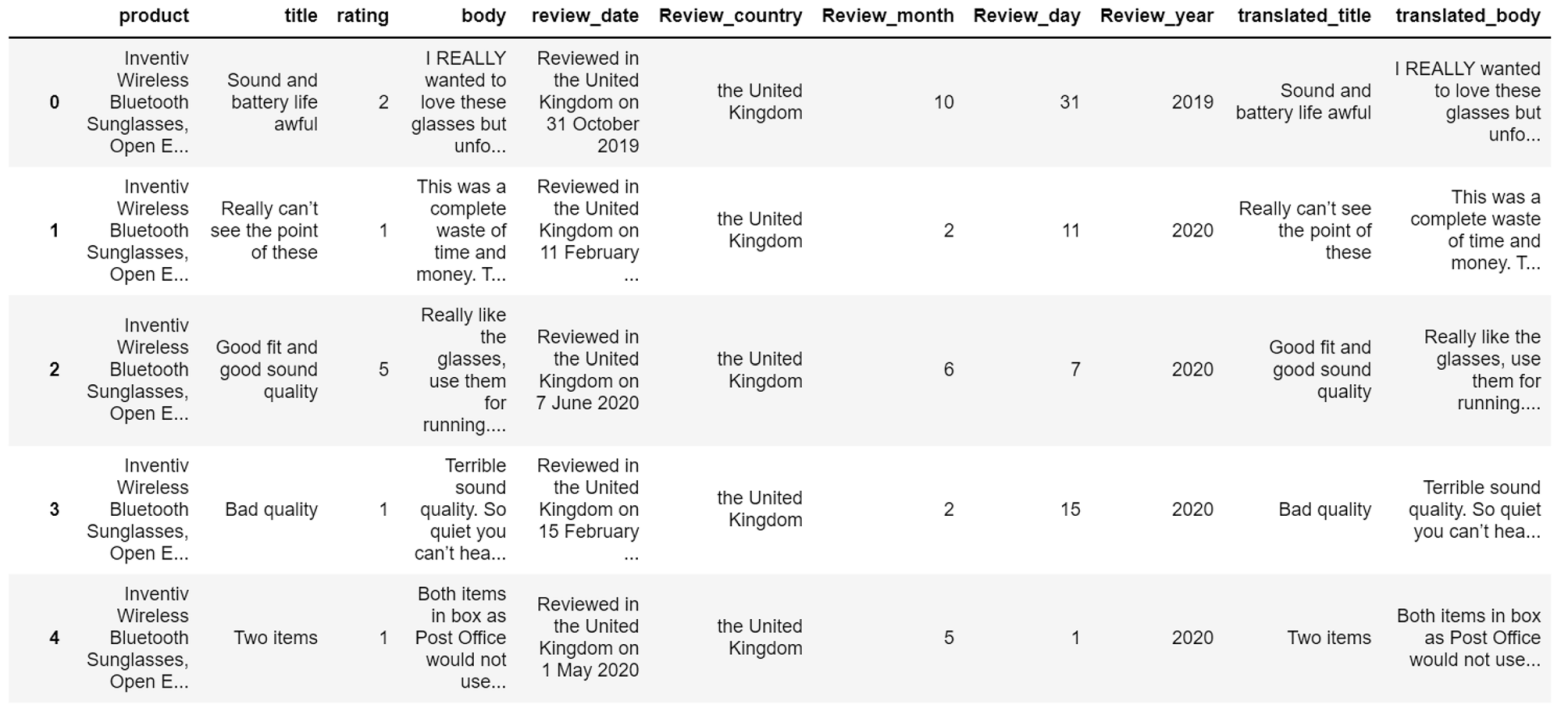
Sample of web scrapping results.

Figure 3 shows the results of the web scrapping. Reviews of the chosen smart glasses’ brands are listed in Appendix A. To further analyse the content of the reviews, Natural Language Toolkit (NLTK) was employed to remove stop words, a total of 245,715 tokens were obtained from the 1997 reviews.

### Deployment of Wordcloud and TextRank algorithm

Since a single review comment usually does not comprise a complete and meaningful paragraph for summarization, the body of the reviews is first ranked by their ratings, then concatenated into a single string or text, and ultimately split to obtain a sentence ranking so as to effectively rank the relationship between each sentence. As a result, the output contains a total of 50 comments, five of which are the most frequently appearing or noteworthy comments in each 1–5-star rating category. This data, viewed as the 'Voice of Customer,' interconnects with the QFD technique to provide valuable feedback related to the conceptual design of smart glasses:

As product reviews on websites are typically short in length, multiple brief reviews are concatenated to form a longer text string to ensure the integrity of the data. The comments are then classified into ten categories based on the five ratings (1–5 star rating). Since each review comment usually does not comprise a complete and meaningful paragraph for summarization, the body of review comments should be firstly ranked by their ratings, then concatenate to a single string or text and ultimately split to obtain a sentence ranking so as to rank the relationship between each sentence. As a result, the output contains a total of 50 comments, five of which are the most frequently appearing or noteworthy comments in each 1–5-star rating category. The data, viewed as the voice of customers, interconnects with the QFD technique to provide valuable feedback related to the conceptual design of smart glasses:

i. 1-star reviews of smart glasses mostly commented on the appearance, battery issues, poor quality, and high cost of smart glasses.
ii. 2-star reviews discussed the poor audio quality compared to other intelligent music devices, showing the preference for headphones among some customers.
iii. 3-star reviews mentioned the sound quality issues as well, with some viewing the glasses as affordable alternatives to luxury items though they still questioned their value. Discussions also included appearance design, materials, and accessories.
iv. 4-star reviews commended the lens quality, comfort, and sound quality, remarking the glasses' lightness and versatility for various occasions.
v. 5-star reviews discussed excellent audio quality and the benefit of polarized lenses for sun protection. One stood out for its superior sound without the bass vibration found in other music glasses.

**Figure 4:**
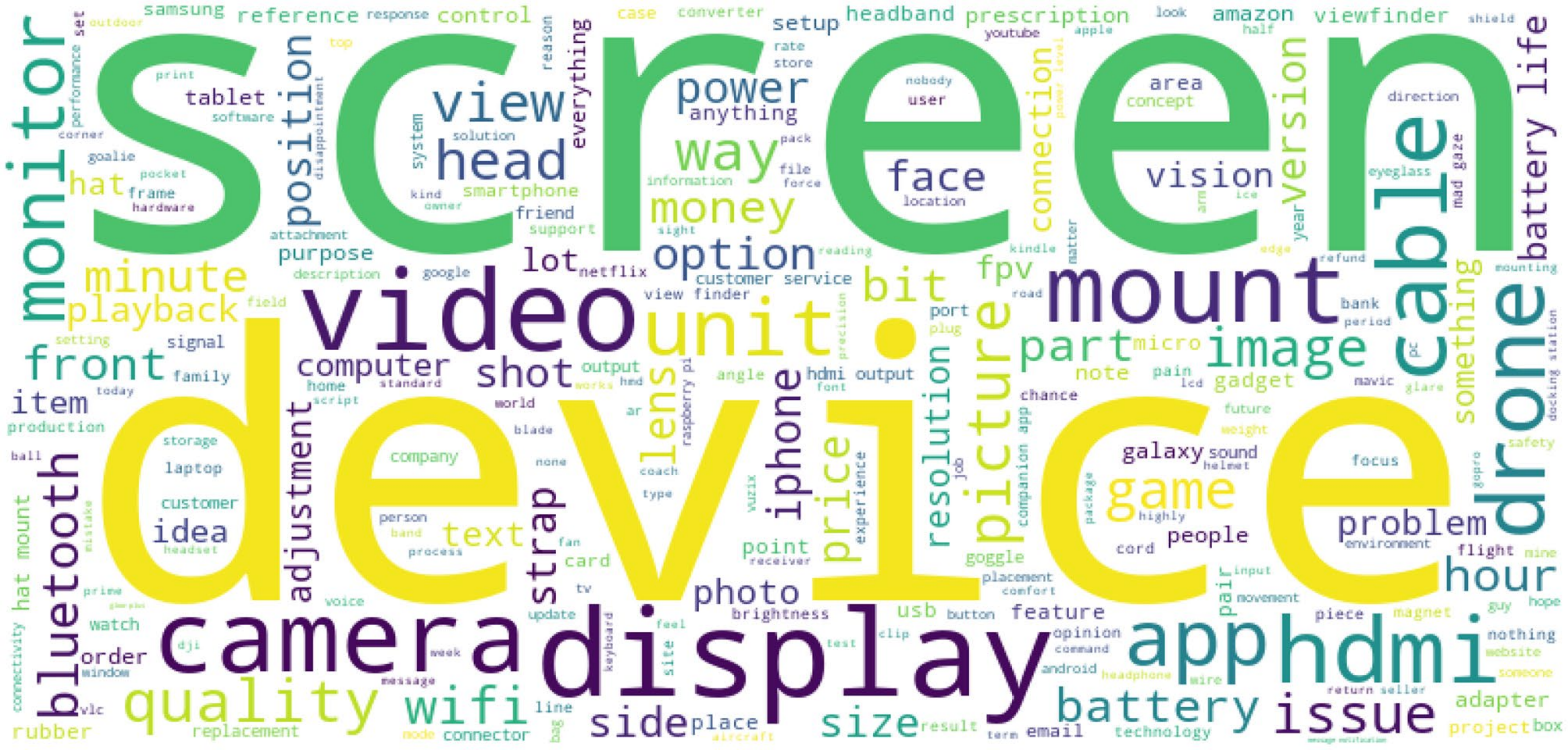
Word cloud of most commonly used phrases in product reviews.

In addition, the most commonly used phrases in smart glasses product reviews are visualized through a wordcloud in Fig. 4 to highlight the main concerns of customers. The size of each keyword in the word cloud corresponds to its frequency in the reviews: the larger the font size of a keyword, the greater the attention that product characteristic receives from consumers. For instance, the largest words like 'screen' and 'display' suggest that these features are frequently mentioned in reviews, which indicates a strong consumer preference for good display resolution and a wide field of view. Appendix A illustrates the findings of five rating levels of comments on smart glasses.

### Deployment of the QFD

Data from the Amazon online comment assists QFD in capturing the voice of the customers and effectively formulating the attributes of customer needs and technique requirements, as shown in Tables 2 and 3. Based on the results of potential customers who give priority to the importance of smart glasses requirements, customers believe that the top three attributes are long battery life, good display resolution, and a wide field of view, which confirm the explicit and implicit needs of customers. Meanwhile, experts rate optical components as the most important technical requirement in smart glasses design, followed by display resolution and battery capacity. Therefore, the optical components are closely linked with customer needs and have high overall weighting.

**Table 2:** The attributes of customer needs.

Customer needs	Description	Importance rate
(C1) Affordable price	A reasonable amount that most people can afford	1.04
(C2) Ease of use	Users can easily understand and operate the product	2.05
(C3) Wide Field of View	The extent of the observable world through the viewing angle	3.85
(C4) Good display resolution	Pixels with a higher resolution provide clarity of visual information	3.98
(C5) Wear comfortably	Thinner and lighter glasses allow people to wear them in a daily routine	2.12
(C6) Long battery life	Power performance can be supported for a certain length of time	4.00
(C7) Lightweight	Less than average compared to most other things of the same type	2.61
(C8) Good sound quality	The audio output (synthesize the accuracy and fidelity) from an electronic device can provide a pleasant experience to users	1.80
(C9) Decent appearance	A well-proportioned look for attractiveness and styling while being socially acceptable	1.98

**Table 3:** The attributes of technique requirements.

Technical requirement	Description	Importance rate
(T1) Appearance design	It forms a visual impression on a viewer, taking into account the shape, color, and material of the smart glasses	1.92
(T2) Volume	It expresses the occupied space of smart glasses, including frames that are open and frames that fold	1.23
(T3) Weight	It expresses the heaviness of smart glasses that are constructed from different materials	3.02
(T4) Battery capacity	It is considered to be the maximum amount of charge that can be stored and support the smart glasses	3.18
(T5) Display resolution	It is considered to be the number of horizontal and vertical pixels on a display screen that affects the visibility of fine and delicate information	3.96
(T6) Total harmonic distortion	It is expressed as the ratio of the sum of all harmonic component powers to the fundamental frequency signal power	1.00
(T7) Processor	It is considered to be the logic circuitry and the basic instructions that respond to driving smart glasses	2.53
(T8) Optical component	It is the construction of optical modules, such as waveguides and birdbaths, that transmit and reflect information to users' visuals	5.00
(T9) Operating procedure	It is considered to be the compiled instructions or methods such as the touchpad, hand gesture, and 3D space control to support user control and carry out routine operations	1.24
(T10) Connectivity	It is considered to be the ability of systems, applications, and platforms to make and maintain a connection with each other, such as Bluetooth, Wi-Fi, NFC, and Type C support	1.57
(T11) Sensors	It is considered to detect events or changes in its environment for interactive functions, such as IMU, camera and LiDar	1.25

**Figure 5:**
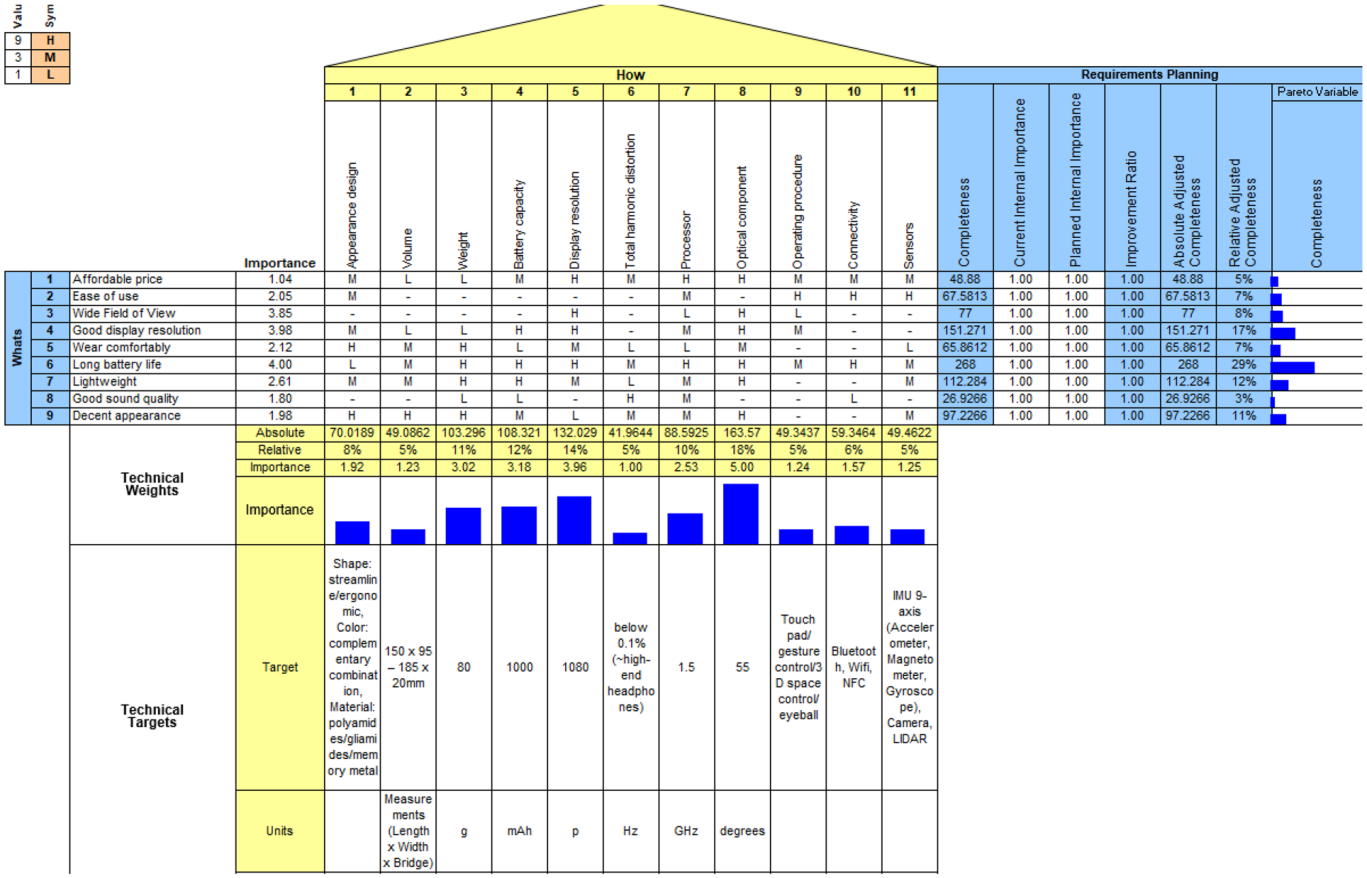
The House of Quality of Smart Glasses.

**Figure 6:**
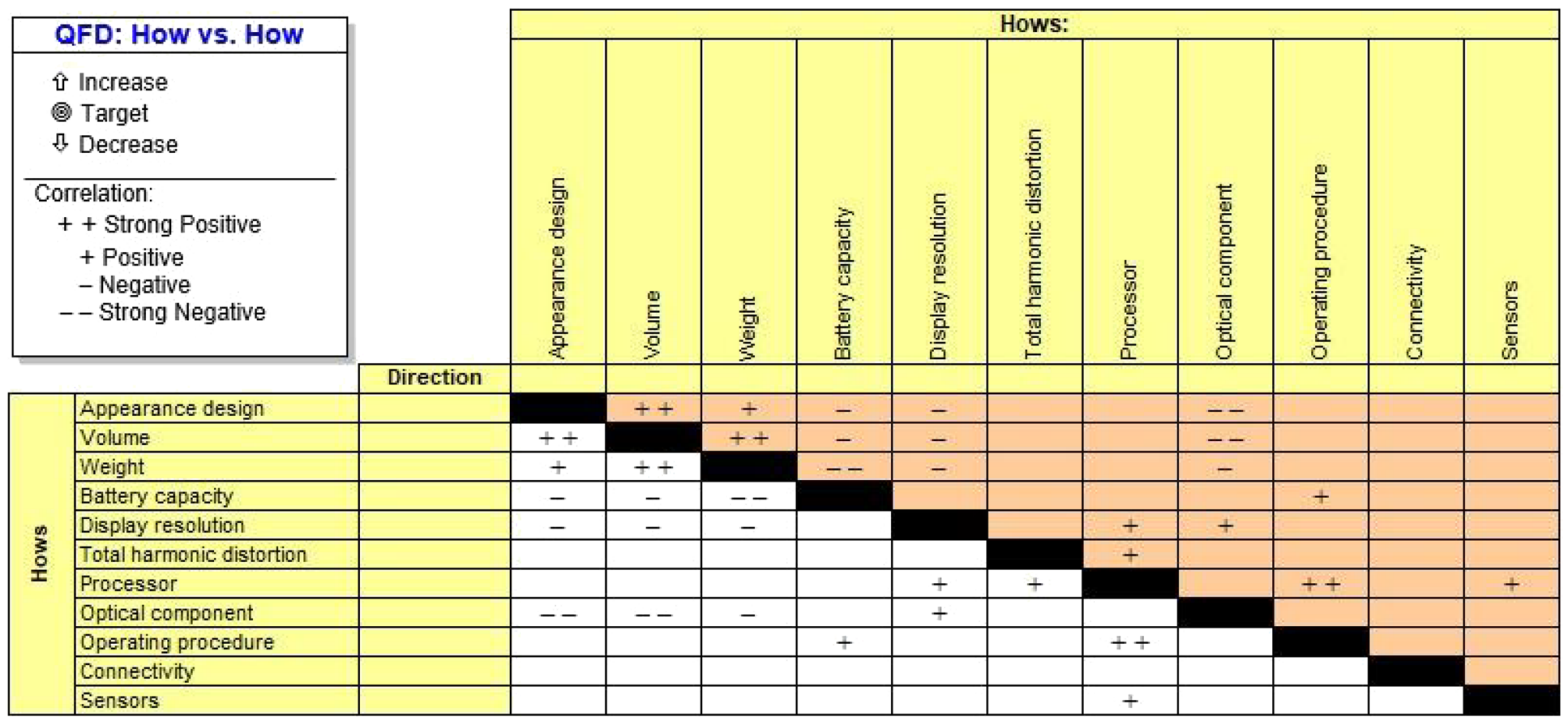
The Interaction Matrix of Technical Requirements of Smart Glasses.

Figure 5 presents the house of quality (HoQ) of smart glasses and Fig. 6 shows the interaction matrix of technical requirements of smart glasses. The findings suggest that the preferable attributes for the next-generation smart glasses should be concentrated on battery life, display resolution, wide field of view, and optical components. This also assists the R&D and design teams in building a clear emphasis in the future. Whereas each smart glass characteristic has a set target value or standard value taken from the relevant standards or opinions of experts to achieve development with technological quality while meeting the customer requirement. The target values of smart glasses are detailed as follows:

- (C1) Appearance design is considered to be a three-element design, shape, color, and material. The shape may be designed to be streamlined and ergonomic. Different colors using complementary colors provide feelings of energy and vitality. The material affects the durability, lightness, and uniqueness of smart glasses. For example, frames made of memory metal are extremely flexible and can be twisted or bent; polyamides and gliamides are strong, lightweight, flexible materials suitable for sports and performance frames.
- (C2) Volume calculated as 150 Length × 95–185 Width × 22 mm Bridge.
- (C3) Weight of 80 g.
- (C4) Battery capacity 1000 mAh.
- (C5) Display resolution 1080p.
- (C6) Total harmonic distortion lower than 0.1% based on the high-end headphones’ standard.
- (C7) Processor speed 1.5 GHz.
- (C8) Optical component has a pitch of 55 degrees.
- (C9) Operating procedure considers a number of aspects, including touchpad, hand gesture control, 3D space control, and so on.
- (C10) Connectivity considers a number of aspects, including Bluetooth, WIFI, NFC, Type C support, and so on.
- (C11) Sensor considers a number of quantities, including IMU 9 axis (accelerometer, magnetometer, gyroscope), camera, LIDAR, and so on.

### Deployment of the TRIZ

Based on the target technical specifications for smart glasses identified through QFD analysis, a combined methodology of TRIZ and Triptych was applied to address the technical contradictions in the design. The analysis revealed that 8 TRIZ inventive Principles, which were generated from the 39 design parameters, closely match the improving and degraded features, including Universality, Periodic Action, Another Dimension, Taking Out, Beforehand Cushioning, Parameter Changes, Self-service, and Mechanics Substitution, as listed in Table 4. These TRIZ principles can be utilized to provide valuable insights for designing the next-generation smart glasses.

**Table 4:** Project ideas generated with TRIZ.

TRIZ Solution	TRIZ contradiction				Project ideas
Principle	Improving feature	Improving feature represents	Degraded feature	Degraded feature represents	
Mechanics Substitution	Weight of stationary object	Weight	Extent of automation	Operating procedure	Utilizing sensory modalities to perform work tasks (e.g. gesture and voice)
Another Dimension	Difficulty of detecting and measuring	Operation procedure	Length of moving object	Volume	Utilizing gesture control with intuitive design
Self-service	Ease of operation	Battery life	Weight of moving object	Weight	Utilizing energy harvested from the human body itself or the environment (e.g., skin charging, sports charging, or solar energy)
Universality	Duration of action by stationary object	Battery life	Weight of the stationary object	Weight	Utilizing the task managers in the smart glasses application to terminate tasks
Periodic Action	Duration of action by stationary object	Battery life	Weight of the stationary object	Display resolution	Adjusting the frames per second (FPS) automatically according to the user behaviour and the display content of smart glasses
Taking Out	Device complexity	Appearance design	Loss of Energy	Operation procedure	Utilizing the modular design for disassembly of all components
Beforehand Cushioning	Reliability	Battery life	Length of stationary object	Appearance design	Utilizing the modular design for adding replacement parts
Parameter Changes	Reliability	Optical component	Loss of Energy	Battery life	Inserting the photochromic lenses and dynamically adjusting to block the external light

**Figure 7:**
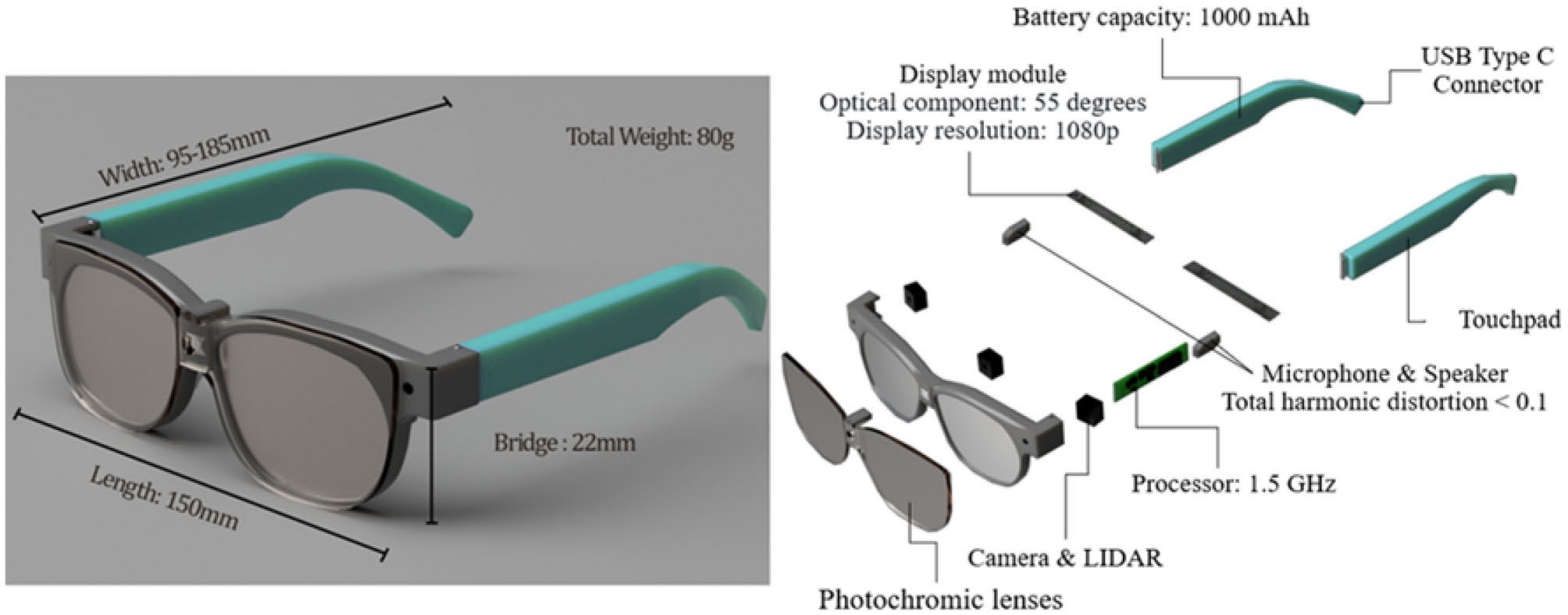
Conceptual design for next-generation smart glasses.

Based on the project ideas generated with Triptych TRIZ above, the concept for smart glasses is shown in Fig. 7. These glasses offer users hands-free digital interaction through a real-time AR display. Designed like traditional eyewear, they weigh only 80 g and are constructed from memory metal and advanced polymers with photochromic lenses that adapt to lighting. The augmented reality experience is enhanced with hand gesture and 3D space control features, facilitated by a 3D LiDAR and a 1080p camera. Voice control is supported by integrated noise-cancelling microphones and speakers, eliminating touchpad use. With a 1.5 GHz processor and a modular 1000 mAh battery, these glasses are designed for optimal performance.

## Results and discussion

The findings from the case study are outlined in response to the research questions introduced in Section "Introduction". Each question is sequentially addressed to provide a structured and comprehensive understanding of the results:

### RQ1: 

How can the customer feedback from digital platforms be identified and analyzed to improve the design of smart glasses?

In our study, a web scraper was developed with Python's Selenium library to collect product reviews on smart glasses from Amazon for analysis. The Natural Language Toolkit (NLTK) was then used for data preprocessing. TextRank algorithm was implemented to rank these reviews by their ratings, which resulted in 50 significant comments from one to five stars rating categories. This feedback, seen as the 'Voice of Customer,' was crucial for the Quality Function Deployment (QFD) process in designing smart glasses. Wordcloud was used to visualize the keywords in customer feedback on smart glasses, which offers a clear picture of customer priorities. These insights into customer preferences and experiences played a key role in guiding the design process towards features that users value the most. By connecting the design with these insights, the lifespan of smart glasses could be extended and contributed to achieving sustainability goals.

### RQ2:

 What are the key factors that influence customer satisfaction with smart glasses, and how can these insights be integrated into QFD and TRIZ methodologies?

Key product features, such as comfort, functionality, and aesthetic appeal, were identified and prioritized in this analysis. Then, these insights were converted into technical specifications using the QFD method. For example, comfort might be translated into specifications for weight and material choices, functionality into user interface design, and aesthetic appeal into the shape and color of the product. Furthermore, the TRIZ contradiction matrix and inventive principles were employed to develop innovative solutions for technical challenges identified in the design process. A typical example of such a contradiction might be balancing the need for lightweight glasses with sufficient battery life and high-resolution displays. Applying TRIZ inventive principles helped find innovative ways to balance these requirements. This methodology, which combines customer feedback with technical innovation, not only enhanced the user experience of the smart glasses but also drove innovation in their design.

### RQ3:

 What is the next-generation smart glasses design based on the latest customer requirements?

The conceptual design of the next-generation smart glasses is elaborated in detail in Section "Deployment of the TRIZ". These advanced smart glasses are characterized by their lightweight design and feature a real-time augmented reality (AR) display. They are equipped with a fast processor and a durable battery, alongside adaptive lenses that respond to varying lighting conditions. For user interaction, the glasses offer intuitive control through hand gestures and voice commands, facilitated by a 3D LiDAR sensor and an integrated camera. Through the combination of these innovative design features, the next-gen smart glasses result in a cutting-edge and sustainable wearable technology.

### Discussion on energy efficiency of smart glasses

The results of case analysis indicates that battery life is the top concern of customers according to the findings through QFD analysis. Smart glasses often require a high energy density and a long battery lifespan to operate various components like displays, cameras, sensors, and wireless connections, etc. Hereby, it is necessary to consider the future development direction of battery development for smart glasses. Two experiments were conducted to evaluate battery durability, which includes investigating the impact of battery power and different levels of camera resolution on smart glasses.

Therefore, it is necessary to consider the future direction of battery development for smart glasses. Two experiments were conducted to evaluate battery durability, which includes investigating the effects of battery power and camera resolution on smart glasses.

**Figure 8:**
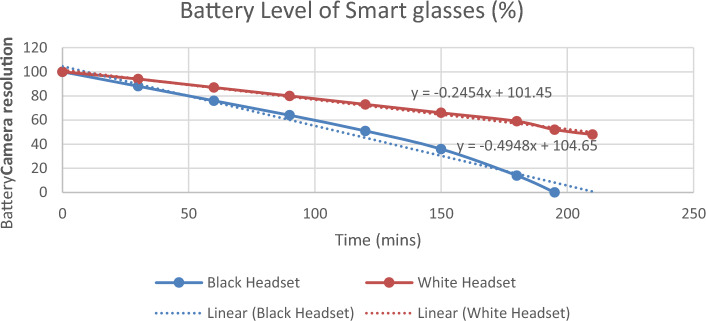
Battery Level of Smart glasses.

One compared two smart glasses batteries: the black headset (ENMESI E633 Head-mounted Smart Glasses) dependent on smartphone chipsets, and the white headset, with their specifications shown in Table 5. Both glasses were set to the lowest brightness and charged to 100% to assess the battery drain rate. The battery level was recorded every 15 min until the battery level drop to 50%. Figure 8 reveals that the battery drain rate of the black headset dropped was twice as fast, with a half-life of 110 min. Whereas the white headset decreased by an average drop of 7% every half hour, with a half-life of 209 min.

**Table 5:** Specification of two smart glasses.

Parameter	Black headset	White headset
Display	Double Screen Display: 1280*720*2PIX	Single LCOS display
Lens	A group of multiple optical resin lenses blue light protection film	Waveguide
Field of view angle	40°	37°
Interface	Type C 3.55 mm Headset 1280*720*2PIX	USB Type c, 3.5 mm headset
Power	1.65W	230 mW @60 Hz

It was found that the lowest resolution (640 × 480) was the most battery-efficient, lasting 116 min to half-battery. In contrast, the highest resolution (1920 × 1080) consumed the most power with a half-life of 104 min. The other resolutions, (1280 × 720) and (1280 × 960), showed similar patterns, depleting 12–13% every 15 min with half-lives of 106 and 108 min, respectively.

**Figure 9:**
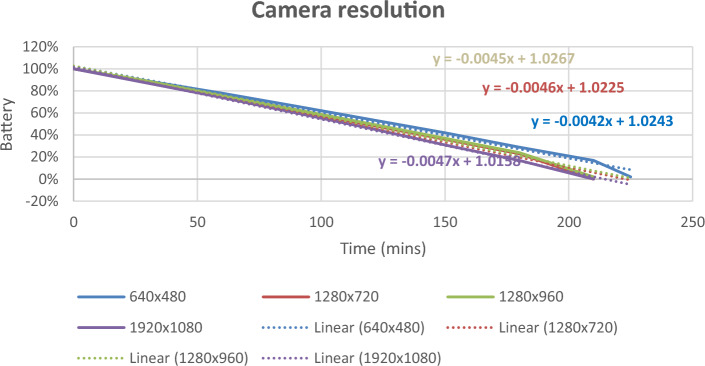
Different levels of Camera Resolution.

The resolution was the most important factor when influencing the energy usage, and there were Another test explored the energy consumption across four different levels of camera resolution. The procedures for this experiment are similar to the previous one. Figure 9 show that the lowest resolution (640 × 480) has the best battery performance, lasting 116 min to half-battery. In contrast, the highest resolution (1920 × 1080) consumed the most power with a half-life of 104 min. The other two resolution levels, (1280 × 720) and 1280 × 960), show the similar patterns, depleting 12 to 13% every 15 min with the half-life of 106 and 108 min, respectively.

The research results above indicate that the battery design of smart glasses should focus on high energy efficiency and long device usage. For instance, it's beneficial to prioritize advanced battery technologies, such as solid-state batteries, lithium-sulfur batteries, and other renewable battery components, to achieve longer usage times between charges and extend the overall battery lifespan. In addition, the integration of renewable or bio-based components in battery production can also reduce the carbon footprint of the manufacturing process. Furthermore, a modular design can be considered as a ground-breaking approach for batteries. It allows users to replace just the drained battery component rather than the entire device, which could effectively extend the device's lifespan, minimize electronic waste, and pave the way for future upgrades without the need for a complete device replacement.

When equipped with energy-efficient components, like optimized cameras that require less power, and user interfaces that guide on power conservation, such design principles can also boost the energy efficiency of smart glasses. These strategies could not only improve the device performance but also play a pivotal role in minimizing greenhouse gas emissions throughout the product's life cycle.

### Discussion on sustainable design of smart glasses

Using the conceptual design of smart glasses products as an example, the feasibility and effectiveness of the proposed methodology are demonstrated. According to the results, eight eco-innovation suggestions have been generated to improve the sustainable design of smart glasses, which can be grouped into three categories:

i. Battery System: It is designed to improve battery longevity, as the high-power consumption of smart glasses and design constraints limit the storage of large batteries. It is based on the Principles of Self-service, Universality, and Periodic Action. Self-sustaining operations, such as harvesting energy from the human body or the environment—like skin charging, sports charging, or solar energy—can be employed as well. Meanwhile, automatically adjusting the frames per second (FPS) of the display content according to need can further increase the battery life of smart glasses, which paves the way for more streamlined designs.
ii. Modularization: Components can be interchanged flexibly to address appearance design, battery life, and optical components, based on the Principles of Taking Out, Beforehand Cushioning, and Parameter Changes. Users can customize their devices according to their preferences, and damaged parts can be replaced individually, thus reducing waste. This design also emphasizes the reusability of products beyond their primary lifecycle.
iii. Sensory Recognition System: Current smart glasses have intricate interfaces, often requiring multiple steps using buttons or touchpads^49^. By applying the Principle of Mechanics Substitution and Another Dimension, these processes can be simplified. Introducing intuitive controls that resonate with users' mental models can help reduce the number of components and boost usability. For instance, while the Nreal AR smart glasses feature a more extensive touchpad, they still miss out on intuitive operations. A shift from traditional buttons or touchpads to sensory interactions, like gestures and voice commands, simplifies user engagement. Such adaptations not only reduce components but also improve the overall user experience.

Each of these categories demonstrates the application of text mining, TRIZ and QFD methodologies in designing smart glasses that are not only technologically advanced but also connect with consumer preferences and sustainability objectives.

## Conclusions

The large amount of waste generated by current consumption patterns causes enormous problems in storage, processing, and disposal space. This study introduces a systematic methodology for sustainable product development. The contribution from this study include:

1. A novel methodology integrating text mining, QFD, and TRIZ has been developed. This framework offers an in-depth. overview of both customer preferences and technical requirements for smart devices, which helps to prevent these smart products from exiting the market and extend their lifespan, aligning with the sustainability goal.
2. Drawing from the vast data in product reviews, insights into consumer preferences for smart glasses were identified. Key features, such as long battery life, exceptional display resolution, and a broad field of view, are vital in consumer choices. Meanwhile, optical components, specific display resolutions, and battery capacity are considered as core elements for design and development teams.
3. Recommendations have been formulated for future product development in the smart device industrybased on analysed consumer feedback and technical evaluations. A roadmap for upcoming innovations in this domain has also been proposed through the identified TRIZ principles. For example, unconventional energy-harnessing solutions, modular design approaches, and a shift to intuitive interaction methods, such as gesture controls, indicate the future direction of smart glasses.

In summary, this study not only addressed the challenges faced in sustainable product design, but also demonstrated the feasibility of an integrated innovation design process based on text mining, QFD, and TRIZ. The proposed approach, pioneering in smart device design, combines customer feedback with QFD and TRIZ methodologies to recommend next-gen smart devices, which indicates the importance of feedback in design refinement and production cost reduction. Limitations of the study include the limited data that may not represent all consumer preferences. Future research is required to expand data sources to include more platforms and consumer reviews from diverse cultural backgrounds for greater diversity. Moreover, investigating the sustainable design and development of other smart products to improve their sustainable product development is also necessary.

### Supplementary Information

Supplementary Information.

## Data Availability

The datasets used and/or analysed during the current study available from the corresponding author on reasonable request.
