# Supplementary material for: Achieving eco-innovative smart glass design with the integration of opinion mining, QFD and TRIZ
Source: Sci Rep. 2024 Apr 29;14:9822. doi: 10.1038/s41598-024-58867-1 (PMC11059285; doi:10.1038/s41598-024-58867-1)
Supplement: Supplementary file 1 — Supplementary Information. [file 41598_2024_58867_MOESM1_ESM.docx]

# Appendix A. The list of smart glasses considered in this study

| **#** | **Brand** | **Product Name** | **Special Feature** |
| --- | --- | --- | --- |
| 1 | Amazon | Echo Frames (2nd Gen) | Hands-free with Alexa, Make calls, Listen to podcasts or Audible books |
| 2 | AOHOGOD | AOHOGOD Smart Audio Bluetooth Sunglasses | UV400 polarized sunglasses of high quality with UV blocking, Bluetooth wireless music speaker |
| 3 | Bear Grylls | Bear Grylls BG-GLS-1 Glasses | Built-in POV camera IP66 Waterproof 90° Sports Camera Impact Resistant, U400 Polarised Lenses |
| 4 | Bose | Bose Frames Tenor | Audio sunglasses, Bose Openaudio, Advanced mic system |
| 5 | Bose | Bose Frames Alto | Open-ear audio, Ultra-modern materials, Bluetooth enabled, UVA/UVB protection |
| 6 | Bose | Bose Frames Soprano | Audio sunglasses, Bose Openaudio, Advanced mic system, Elegant cat eye |
| 7 | Cinemizer | Cinemizer OLED Video Glasses | Video glasses for movies watching, A variety of devices from smartphones with HDMI connectivity, Integrated rechargeable battery |
| 8 | DZQH-Qin | DZQH-Qin Wireless Bluetooth Sunglasses | Wireless for listening to stereo music, Bluetooth connectivity |
| 9 | Epson | Moverio BT-300 | Transparent Si-OLED display, Applications of photography or aerial inspections, High-resolution front camera |
| 10 | EUNY | AS6 VISION-800 | Bluetooth and wifi connectivity, 5.0MP camera, able for downloading 3D format videos, interactive gaming experience with mouse, keyboard, gamepad connection support |
| 11 | Eye Connect | Eye Connect Bluetooth Sunglasses | Bluetooth enabled iOS/Android, Compatible with smartphones to make hands free calls and listen to music |
| 12 | FAUNA | Fabula Crystal Brown | Touch sensor in both temples, Two microphones with beamforming algorithm, fashion glasses |
| 13 | GELETE | A12Pro | Open ear bluetooth speaker, UV400 polarized lens, IPX4 waterproof sunglasses, Calls/music/navigation, Bluetooth connectivity |
| 14 | GoVision | GoVision SOL | 5MP CMOS sensor, 63-degree wide-angle view, 15MP camera resolution, Up to 3 hours of music playtime |
| 15 | Guangtouqiang | Bluetooth Audio Glasses | Bluetooth audio glasses, Noise cancelling ,mic, Open ear music headset |
| 16 | Helalife | Helalife Smart Sunglasses | Wireless Bluetooth Audio, Calling and llistening to music, Suitable for outdoor usages, Waterproof, Lightweight, Long standby time |
| 17 | Hereta | Hereta Wireless Bluetooth Sunglasses | Anti-ray stereo 4.1 music bluetooth headphones, Hands-free for smartphones |
| 18 | HYFILOD | Smart Glasses | Polarized sunglasses with bone conduction, Bluetooth connectivity speaker, Open-ear audio |
| 19 | Inventiv | Inventiv Bluetooth Sunglasses | Wireless, Microphone, Sports-and-exercise, USB connectivity, IOS, Android |
| 20 | Inventiv | Inventiv Classic Bluetooth Sunglasses | Bluetooth audio glasses, Open ear headphones, Hands free calling |
| 21 | Inventiv | Inventiv Sport Bluetooth Sunglasses | Wireless, Sports-and-exercise, USB connectivity, IOS, Android |
| 22 | ISCREM | ISCREM Video Camera Sunglasses | HD video recording, Long-time video recording, Outdoor sport |
| 23 | Kaleser | Kaleser Spy Hidden Camera Glasses | Built-in HD hidden camera, Video quality of 1920*1080p HD Resolution and 140-degree viewing angle |
| 24 | KENTKING | KENTKING Smart Glasses | Open ear audio, Full range of outdoor activities usages |
| 25 | KTYXGKL | KTYXGKL Smart Audio Glasses | Bluetooth Compatible, Directional audio technology, Open ear speaker system |
| 26 | Leaden | Leaden Wireless Bluetooth MP3 Sunglasses | Bluetooth headphones for driving, cycling and hiking, Foldable eyeglasses design with freely telescoping boom speakers 270 degree rotatable |
| 27 | Lucyd | Lucyd Lyte | Open-ear audio, Crisp microphone, Bluetooth touch controls, Fashionable and prescription-ready frames |
| 28 | MAD Gaze | Glow Plus | Optics of BirdBath with OLED display, 1080p resolution, 53° (Diagonal) FOV, Hand gesture control, Voice recognition |
| 29 | MAD Gaze | Vader | Game playing, Video watching, Large screen at 45 degrees FOV |
| 30 | Monkaim | Monkaim Spy Glasses | Hidden camera glasses, One-button operation, Looping record and card storage |
| 31 | OhO sunshine | OhO Bluetooth Sunglasses | 4K pro ultra HD video recording, Impact resistant lenses and frame, One touch button operation |
| 32 | Panyihan | Smart Audio Sunglasses | Bluetooth Compatible, UV400 protection, IP67 waterproof and dustproof performance |
| 33 | PEGASI | PEGASI 2 | Wavelengths of light in solving sleep problems, Ease of use, Lightweight |
| 34 | RAYCORE VIEW | Bluetooth Wireless Smart Glasses | Music glasses with open-ear speakers, Touch control headphone, Integrated with microphone |
| 35 | SunnyCam | SunnyCam Active | Vibrate alert and 1 hours of full HD video recording time, Slim design with fully interchangeable lenses |
| 36 | TechKen | TechKen Sunglasses | Bluetooth wireless music headset with polarized lenses, Comfortable wearing and secure for running/hiking |
| 37 | TJ | TJ Half frames | Open ear headphone, Voice control, Phone call, Music player, Sports |
| 38 | Vocalskull | Vocalskull Alien5 | Bluetooth Compatible, Bone conduction, Make calls, Listen to music |
| 39 | Vufine | VUF-110 | High definition virtual display, HDMI compatible |
| 40 | Vuzix | Vuzix Blade | Autofocus HD camera, Integrated stereo speakers with noise-cancelling mics, Full color right eye display, Full UV protection lenses with ANSI Z87.1 safety certification, Wireless Wi-Fi and bluetooth |
| 41 | WGP | WGP Smart Audio Sunglasses (2nd Gen) | Open Ear Speaker, Bluetooth Connectivity |
| 42 | WGP | WGP Smart Audio Glasses | Outdoor cycling sport glasses, Anti-blue light lens, Open ear speaker with bluetooth connectivity |

# Appendix B. Results of applying TextRank in term of rating 1 to 5

Rating 1:

| 1 | Glass wiping cloth isn't provided with this unit. |
| --- | --- |
| 2 | Returned glasses, looking for alternative to recording work projects. |
| **3** | **Battery on the frame of glasses was about to explode from outdoor heat.** |
| **4** | **Glasses are light, trendy and the sound is pretty decent.** |
| **5** | **When I put the glasses ABOVE my ears (= the normal way to wear glasses), then the image on the right side is blurry.** |
| 6 | Like reading glasses bought from the Supermarket, pretty low quality as you would expect from the price. |
| 7 | I paid Â£35. 99 for glasses and usb stick and sd card only got the glasses which are only Â£29. 99 ? |
| 8 | I imagine most of us already have echo devices throughout our homes, so why wear glasses to give echo commands or ask it questions? |
| 9 | **Waste of money, and failed to function even as glasses frames due to the incorrect lenses included.** |
| 10 | **Lastly, I was unable to place view finder directly in front of my eye (the attachment for glasses did not alow the view finder to align with my eye)** |

Rating 2:

| 1 | AM radio frequency quality, making vocal tracks too high end. |
| --- | --- |
| 2 | **Summary: I wish I could say these are great glasses since the price is so low** |
| 3 | **But music is not going to be enjoyable, especially if you want great music sound quality.** |
| 4 | **My recommendation. save the money if you are looking for a pair of glasses to hear music, as there are more superior products out there.** |
| 5 | But just for glasses they're good. |
| 6 | Update a week later, glasses charged full and they have stopped working. |
| 7 | Totally charged yet glasses won't turn on. |
| 8 | Unfortunately as others have noted, these are glasses with **mini-speakers, not bone conduction.** |
| 9 | I have **returned them and will buy decent headphones and glasses.** |
| 10 | For who it may concern, First time I contacted Amazon 6 months after purchasing my first pair of glasses, when the coating layer started peeling off. |

Rating 3:

| 1 | I had previously sampled Bose glasses at Walmart which were awesome (to be fair these are different technology, keep that in mind) and I believe my needs to be better met by a product like that. |
| --- | --- |
| 2 | **Bose glasses have a very beautiful design for me, the construction materials are of the highest quality.** |
| 3 | **I love the concept but wish we could buy good quality sound without paying $150+.** |
| 4 | **Great glasses only downside is the sound quality not Bose but still much cheaper alternative.** |
| 5 | Nice looking glasses but not worth the money for the sound quality. |
| 6 | **The sound is "Tin"-ny but ok, not great sound, but maybe would be a good value at half the price due to the poor quality sound but as glasses they are great** |
| 7 | **It's a well made pair of glasses, it comes with a nice bag and charging cord, and seemed to have polarized lenses.** |
| 8 | These glasses look chunky and remind me of some cheap pair of glasses off the market. |
| 9 | So I'm listening to music and then decide to temporarily put the glasses down and as a wearer of glasses, I put them down upside down as I always do. |
| 10 | I did not know there were two quantities of glasses. |

Rating 4:

| 1 | **Good glasses and comfortable wear amazing sound.** |
| --- | --- |
| 2 | The lenses are of decent quality and although not exactly up to the quality of something like Persol, I would easily put them at Ray-Ban quality of glass lenses. |
| 3 | The lenges don't smudge as easily as other glasses I wear and are great sun protection. |
| 4 | Lots of other glasses are too fancy for my taste and may draw the attention of robbers. |
| 5 | **The seat is Firm, but not oppressive, and due to its low weight it ultimately fits just like other glasses.** |
| 6 | A microfiber cloth keeps the glasses clean, just as I know it from other glasses. |
| 7 | I think it's cool that you can have other glasses set at the opticians. |
| 8 | **On the visual side they are great glasses.** |
| 9 | In short, for me they are great glasses, with a model that combines with all types of wardrobe, ranging from casual, semi-sporty or elegant/formal but with the condition that I cannot use them all the time or every day at least. |
| 10 | All in all, it is really great glasses, but unfortunately with deductions for me. |

Rating 5:

| 1 | Providing **crystal clear audio** that immerses the listener within the soundscape, at the same time they are good quality glasses with **polarized lenses and with UVA / UVB protection** (therefore from excellent level of protection) |
| --- | --- |
| 2 | 4th different model of music glasses I've owned now. |
| 3 | I can feel about a **30-40% increase in sound performance** from the speakers and the best part is they **don't have the annoying bass vibration** like other music glasses do. |
| 4 | **I bought them in promotion, they are quality glasses, comfortable, with good bose sound, I recommend your purchase.** |
| 5 | Best glasses with camera to buy! |
| 6 | Great glasses / great **sound quality** / fits perfectly. |
| 7 | Great glasses, **sounds** are great even if it's just in your hair. |
| 8 | **Great glasses, easy to use, decent sound for sunglasses I have the blue light filter glasses because I want to be able to put them on even on dark days.** |
| 9 | **I am amazed on the sound quality over Bose glasses.** |
| 10 | **What is also very important for me is that they support prescription lenses as I am wearing prescription glasses, I wouldn't be able to use Bose glasses daily without changing the lenses.** |
